# Differential responses to con- and allospecific visual cues in juvenile ravens (*Corvus corax*): the ontogeny of gaze following and social predictions

**DOI:** 10.1007/s10071-023-01772-3

**Published:** 2023-04-07

**Authors:** Claudia Zeiträg, Mathias Osvath

**Affiliations:** Department of Philosophy and Cognitive Science, Lund University, Helgonavägen 3, 22100 Lund, Sweden

**Keywords:** Gaze, Ontogeny, Social cognition, Social predictions

## Abstract

Gaze following refers to the ability to co-orient with others’ gaze directions. Ontogenetic studies on gaze following in animals have predominantly used human experimenters as demonstrators. It is, however, likely that developing animals are initially more attuned to individuals from their own species, which might result in differences in the ontogenetic onset of gaze following with human and conspecific demonstrators. “Checking back” is a signature behaviour in the gaze following repertoires of humans, apes, and some Old world monkeys. It is commonly interpreted as a representation of the referentiality of gaze and is thus diagnostic of social predictions. Recently, “checking back” has been discovered in four avian species, suggesting a shared skill among birds. To investigate effects of con- and allospecific demonstrators on gaze following responses, we studied visual co-orientations of four hand-raised juvenile common ravens (*Corvus corax*) with human and conspecific gaze cues. Moreover, we for the first time investigated “checking back” in ravens and compared the effects of con- and allospecific demonstrators on this behaviour. Ravens followed human and conspecific gaze with no apparent differences in ontogenetic onset, but after significantly longer latencies with human demonstrators. Subjects moreover already checked back at 30 days old and did so significantly more often with conspecific demonstrators. Our findings suggest differences in processing speed and social predictions of human and conspecific gazes, indicating an underlying neurocognitive mechanism attuned to social information gathering from conspecifics. We propose more studies using conspecific demonstrators to reveal the full gaze following potential of a species.

**Supplementary Information:**

The online version contains supplementary material available at 10.1007/s10071-023-01772-3.

## Introduction

The transfer and use of social information is an integral part of sociality (Shettleworth 2010). One effective way of acquiring such information is to attend to what others are looking at. Co-orienting with others’ gaze directions (gaze following) is a fundamental socio-cognitive component of human as well as non-human animals. The advantages of extracting social information from observed gaze range from gathering information about food and predators, to drawing attention to social interactions (Emery 2000; Tomasello et al. 1998). Witnessing others’ social interactions can subsequently inform animals about third-party relationships and facilitate social learning.

Human infants are already as new-borns sensitive to others’ gaze directions (Batki et al. 2000; Farroni et al. 2002) and spontaneously start co-orienting with gazes between 3 and 6 months (e.g., Butterworth and Jarrett 1991; Perra and Gattis 2010). The early ontogeny of gaze following in humans illustrates the fundamental character of this socio-cognitive skill that subsequently has implications for the development of other cognitive capacities. In human children, for example, gaze following affects the development of theory of mind (Brooks and Meltzoff 2015), joint attention (Carpenter et al. 1998), and language acquisition (Baldwin 1991; Houston-Price et al. 2006; Schafer and Plunkett 1998).

Many similarities in the development of gaze following in human infants and young animals have been discovered. Co-orientations with observed gaze directions develop early in the ontogeny of mammals [e.g., wolves (*Canis lupus*): Range and Virányi 2011; rhesus macaques (*Macaca mulatta*) and chimpanzees (*Pan troglodytes*): Tomasello et al. 2001] and birds [ravens (*Corvus corax*): Bugnyar et al. 2004; greylag geese (*Anser anser*): Kehmeier et al. 2011; rooks (*Corvus frugilegus*): Schloegl et al. 2008].

However, it is difficult to draw parallels between developmental studies on humans and animals, as human infants are tested for their ability to follow conspecific gaze, while most animals, so far, have been presented with gaze cues from an allospecific demonstrator—a human experimenter. While this practice is beneficial to keep testing conditions as controlled as possible, gaze following has presumably evolved to facilitate the transfer of social information between conspecifics. Therefore, animals are likely initially more attuned to social signals from conspecifics. Animals might learn to interpret human communicative signals later in their development given enough exposure to humans. Parent-raised orangutans (*Pongo pygmaeus*), for example, fail to use human gaze to locate a target (Byrnit 2004) and only enculturated chimpanzees (*Pan troglodytes*) are sensitive to visual attentive states of a human experimenter (Call et al. 2000). Consequently, animals might develop the ability to follow human gaze later than conspecific gaze. Only one observational account for such a disparity exists for ravens (Schloegl et al. 2007). The authors observed ravens co-orienting with their conspecifics’ gazes shortly after fledging—approximately 7 weeks before reacting to experimental human gaze cues. That study, however, did not experimentally compare the ravens’ reactions to human and conspecific gaze cues.

Human children develop into increasingly skilled gaze followers throughout their ontogeny. At 8 months, children begin to look back at a demonstrator when following their gaze and not findings anything interesting in their line of sight (Butterworth and Cochran 1980; Scaife and Bruner 1975). Developmental psychologists commonly view this “checking back” behaviour as diagnostic of an expectancy violation: the failure of finding something in the environment that the gazer was expected to look at. Hence, the behaviour reveals a representation of the referentiality of a gaze (Okamoto-Barth et al. 2007). It is thus diagnostic of the formation of predictions about others’ visual perspectives and behaviours. The comparably late ontogenetic onset of “checking back” compared to co-orientations further points towards and involvement of these more complex neurocognitive mechanisms.

“Checking back” has later also been observed in apes (Bräuer et al. 2005; Horton and Caldwell 2006; Okamoto-Barth et al. 2007) and some Old World monkeys (Goossens et al. 2008; Scerif et al. 2004), while still not shown in New world monkeys (Amici et al. 2009). Recently, this behaviour has for the first time been described in birds, namely three palaeognath species (emus, *Dromaius novaehollandiae*, greater rheas, *Rhea americana*, and elegant-crested tinamous, *Eudromia elegans*), and one neognath species (red junglefowl, *Gallus gallus*) (Zeiträg et al. 2022, Preprint). These new findings raise the possibility of “checking back” behaviour being a conserved behavioural trait among all birds, though it has to date never been described in any other avian species.

Developmental accounts of “checking back” in animals are hence scarce, though, at least in apes, it appears to follow the same developmental pattern as in human children. Bräuer and colleagues (2005) found that in all four ape species, infants (1–4 years) did not “check back” with the demonstrator, but started to show this behaviour as juveniles (5–10 years), and were most likely to “check back” as adults (10+ years).

To obtain a better understanding of the impact of con- and allospecific demonstrators on gaze following responses of developing animals, we tested four hand-raised juvenile common ravens (*Corvus corax*) for their ability to follow human and conspecific gazes into the distance between the age of 30 and 95 days. We moreover investigated “checking back” in ravens, its ontogenetic development, and the potential effects of different demonstrators on this behaviour.

## Methods

### Subjects and housing

We tested four hand-raised juvenile ravens of unknown sex that came from three different (captive) nests, i.e., two of them were siblings. Testing was carried out at Lund University Corvid Cognition Station. All four chicks were initially kept together in an artificial nest, where they were cared for by humans. They were ringed for individual recognition. After fledging, the ravens were moved to an outdoor aviary section of 240 m^2^, which was also shared with two adult females, unrelated to the chicks. They were continually hand-fed by humans (and by one of the females), until they could provide for themselves. Time of fledging was used to estimate the age of two ravens; the age of the two siblings was known. The subjects were 30, 38, and 44 days old at study onset. They had been taken into human care at 13, 20, and 17 days of age and had thus been fed and cared for by humans from an early age.

### Experimental design

The experiment was divided into two demonstrator conditions: a human and a conspecific condition. Each condition further consisted of two trial types: control and test trials.

In the human condition, a human demonstrator was standing or kneeling approximately 2 m in front of the subject, so that they were on eye level. At the beginning of each trial, the human demonstrator caught the subject’s attention through calling or waving. The trial started once the subject’s beak was pointing towards the human demonstrator. In control trials, the human demonstrator looked for 5 s in the direction of the subject, without directly looking at it. In test trials, the human demonstrator gazed up for 5 s through lifting both head and eyes. Two familiar human demonstrators were used that were both involved in hand-raising the ravens.

**Fig. 1:**
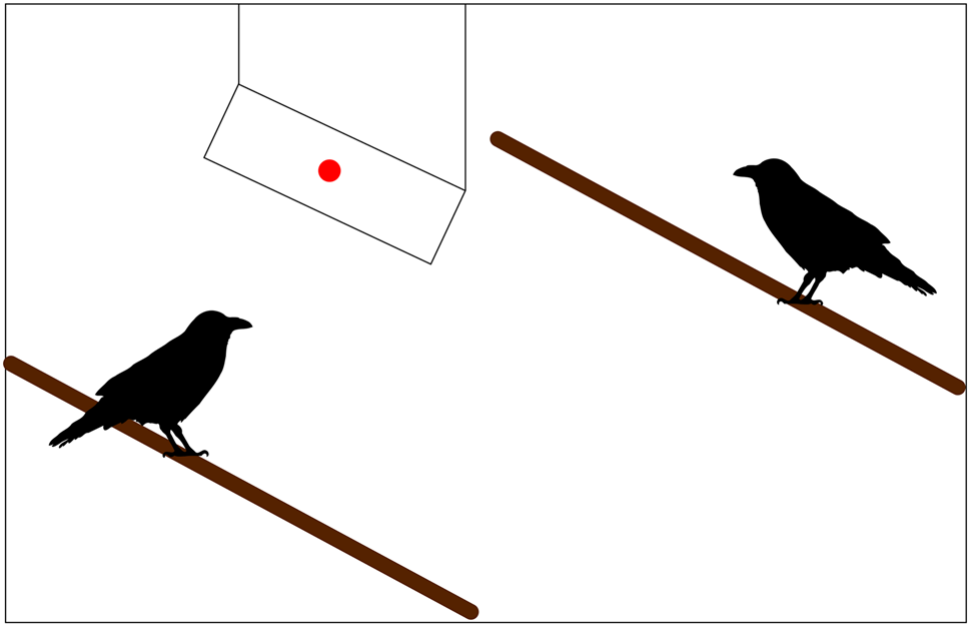
Schematic experimental setup of conspecific test trials. The bird on the left represents the demonstrator; the bird on the right the subject. The red dot represents the gazing stimulus produced by a laser pointer that was used to lure the demonstrator bird’s gaze upwards. In conspecific control trials, no laser pointer was used, so that the demonstrator was not giving a gaze cue. In stimulus control trials, the experimental setup was the same, but no demonstrator bird was present

In the conspecific condition, two ravens were placed on perches facing each other approximately 2 m apart. The experimenter waited for a moment when both birds faced each other, i.e., when their beaks were pointing towards each other, before starting a trial. In test trials, the experimenter lured the gaze of the demonstrator bird to a board hanging above the birds’ heads. This was achieved by reflecting the beam of a laser pointer onto the board on the demonstrator bird’s side until the demonstrator bird reacted by looking up (see Fig. 1). In control trials, no stimulus was flashed, so that the birds were just facing each other. Subjects served as demonstrators for each other. Demonstrators were assigned randomly.

In all trials of both demonstrator conditions, the reaction of the subject was recorded for 10 s after the demonstration in test trials or for 15 s in control trials. To control whether the laser pointer beam was visible to from the subject’s side, we added a third trial type to the conspecific condition: stimulus controls. These controls were conducted in the same way as conspecific test trials, but without a demonstrator present, so that only the beam of the laser pointer was flashed without a gaze cue.

All five trial types (human test, human control, conspecific test, conspecific control, and stimulus control) were pseudorandomized for each subject, as was the order of tested subjects. For examples of each trial type, see videos in the Supplementary Material.

The experiment was run for 8 weeks, with two experimental sessions per week in the first 3 weeks and one session per week for the remainder of the experimental period to reduce habituation. We moreover had to stop conspecific trials after 3 weeks, as it became too difficult to engage two juvenile ravens in the experiment, due to higher mobility and increased exploratory behaviours. Each session took place between 8 a.m. and 3 p.m. with many breaks in between due to the birds’ inability to sit still for longer periods of time and sleeping bouts. Sessions were flexibly adapted to the birds’ propensity to participate in the experiment.

We attempted to execute two trials of each trial type per session, resulting in ten trials per day and subject. This goal was, however, not always met, especially later in the experimental period when the subjects’ interest in exploring and playing grew. For information on the numbers of trials and subjects on each day, see Table 1 (for more detailed information, see Supplementary Material Table 1). All trials were video recorded with two cameras.

**Table 1:** Trials conducted on each experimental day

Date	Age [days]	Demonstrator condition	Trial type	#Trials
210512	30,37,44,44	Conspecific	Test	8
210512	30,37,44,44	Conspecific	Control	8
210517	35,42,49,49	Human	Test	7
210517	35,42,49,49	Human	Control	7
210517	35,42,49,49	Conspecific	Test	6
210517	35,42,49,49	Conspecific	Control	6
210521	39,46,53,53	Human	Test	8
210521	39,46,53,53	Human	Control	8
210521	39,46,53,53	Conspecific	Test	8
210521	39,46,53,53	Conspecific	Control	8
210524	42,49,56,56	Human	Test	8
210524	42,49,56,56	Human	Control	8
210524	42,49,56,56	Conspecific	Test	8
210524	42,49,56,56	Conspecific	Control	8
210524	42,49,56,56	Conspecific	Stimulus control	7
210528	46,53,60,60	Human	Test	8
210528	46,53,60,60	Human	Control	8
210528	46,53,60,60	Conspecific	Test	8
210528	46,53,60,60	Conspecific	Control	8
210528	46,53,60,60	Conspecific	Stimulus control	8
210531	49,56,63,63	Human	Test	6
210531	49,56,63,63	Human	Control	5
210531	49,56,63,63	Conspecific	Test	3
210531	49,56,63,63	Conspecific	Control	4
210531	49,56,63,63	Conspecific	Stimulus control	7
210607	56,63,70,70	Human	Test	8
210607	56,63,70,70	Human	Control	8
210607	56,63,70,70	Conspecific	Stimulus control	8
210614	63,70,77,77	Human	Test	8
210614	63,70,77,77	Human	Control	8
210621	70,77,84,84	Human	Test	4
210621	70,77,84,84	Human	Control	4
210628	77,84,91,91	Human	Test	5
210628	77,84,91,91	Human	Control	4
210702	81,88,95	Human	Test	2
210702	81,88,95	Human	Control	1
Total		Human Human Conspecific Conspecific Conspecific	Test Control Test Control Stimulus Control	64 61 41 42 30

### Coding and statistical analyses

All trials were coded from video recordings using the program Solomon Coder (Version: beta 19.08.02; Péter 2017). We defined the upward gaze of the demonstrator (human or conspecific) or the experimenters signal to start a trial (marked by a vocal signal of the experimenter) in controls as starting point to code trials. From this starting point on, we coded upward looks of the subject, including latency and duration of these orientations. Upward looks were inferred from beak orientations. We moreover coded “checking back” every time a subject looked back at the demonstrator after looking up. The latencies and durations of this behaviour were also coded. For numbers of behaviours identified in this way, see Table 2. Ten percent of the videos were coded for inter-observer reliability and intraclass correlation was excellent (ICC = 0.95, *F* = 36.3, *p* < 0.001).

**Table 2:** Number of occurrences of looking up and “checking back” per trial type

Behaviour	Demonstrator condition	Trial type	Number of occurrences
Looking up	Human	Test	27
	Human	Control	15
	Conspecific	Test	24
	Conspecific	Control	9
	Conspecific	Stimulus control	10
“Checking back”	Human	Test	14
	Conspecific	Test	21

To analyze the ontogenetic onset of gaze following behaviours despite only two subjects having the same age, we divided the subjects into ranges of 10 days starting at day 30. This resulted in seven age ranges: 1 (30–40 days), 2 (41–50 days), 3 (51–60 days), 4 (61–70 days), 5 (71–80 days), 6 (81–90 days), and 7 (91–95 days). For example, on the first testing day, two subjects were in age range 1 (30–40 days) and two in age range 2 (41–50 days).

Generalized linear mixed models (GLMMs) were used to analyze the data with the glmer function of the lme4 package in RStudio (Version 1.4.1717; RStudio Team 2020). To explore the effects of different fixed variables, we created a full model using demonstrator condition (human or conspecific), age, and trial type (test, control, and stimulus control), as well as their three-way interaction as fixed effects and upward looks as dependent variable with a binomial distribution. We included subject as random factor. We ran the same model with latency of looking up as response variable with a Gamma distribution. When using latency as response variable, it was transformed by adding 1 to each value to avoid errors due to zero values in the data. We ran a third GLMM analysis with the same fixed effects and random factor, but “checking back” as response variable with a binomial distribution.

To analyze the ontogeny of gaze following responses, we ran a separate GLMM analysis for each pre-defined age range using demonstrator condition and trial type, as well as their two-way interaction as fixed effects and upward looks as dependent variable with a binomial distribution. Again, we included subject as random factor.

Due to the discovery of an effect of demonstrator condition on the latency of gaze following responses, we re-ran the first model (fixed effects: demonstrator condition, age, trial type, their three-way interaction; dependent variable: upward looks; random factor: subject), but with a dataset excluding all co-orientations that happened more than 5 s after the demonstration. Subsequently, we ran the same model two more times, but for each demonstrator condition separately.

All full models were subsequently reduced with the aim of identifying the best-fitting model using the Akaike Information Criterion (AIC). Fixed factors were excluded from the models if the AIC of the model without that factor was more than two points lower than the full model. This was done with the drop1 function. Consequently, only factors explaining variance were kept in the final models. The effects of the remaining factors were determined through likelihood ratio tests (for values of final models, see Supplementary Material Table 4).

## Results

**Fig. 2:**
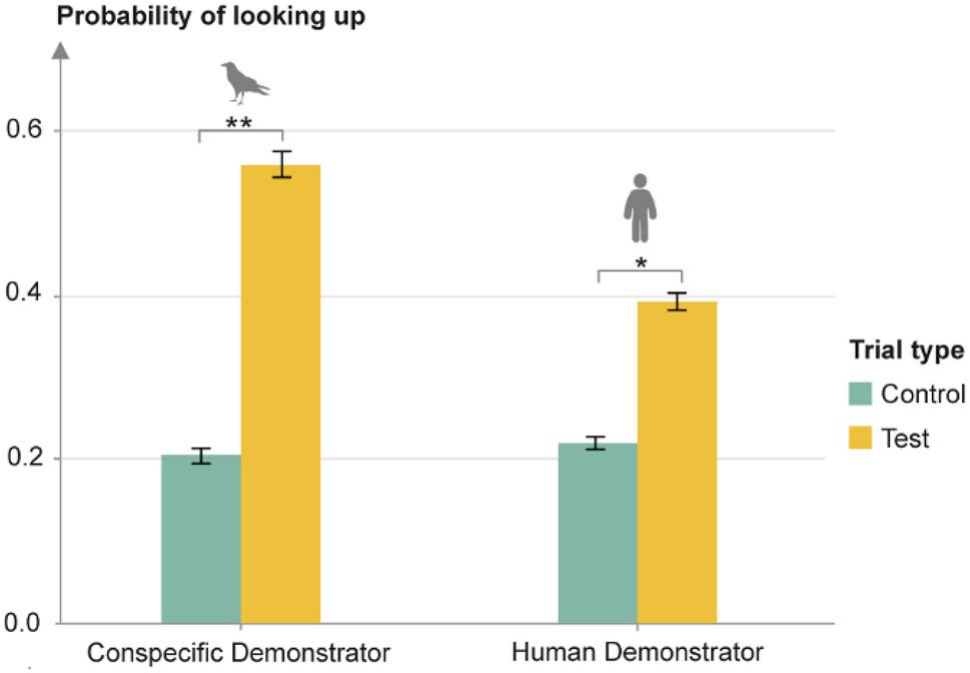
Probability of looking up in control compared to test trials with conspecific and human demonstrators

When analyzing experiments throughout the entire experimental period, we found a significant effect for trial type (test or control; GLMM; *χ*^2^ = 11.95, d*f* = 2, *p* = 0.0025, see Fig. 2), but not for demonstrator condition (human or conspecific) on upward looks. The final model explained more variance than a model including age (Δ AIC > 10). Thus, no developmental trend over the course of the experimental period could be identified. Ravens looked up significantly more often in conspecific test trials compared to stimulus control trials (GLMM; *χ*^2^ = 5.57, *df* = 1, *p* = 0.018), suggesting that they could not see the beam of the laser pointer, but that it was the gaze of the demonstrator that caused the upward orientation.

When categorizing subjects into age ranges of, respectively, 10 days, a first significant effect of trial type (test or control) was identified at 51–60 days (GLMM; *χ*^2^ = 6.31, *df* = 2, *p* = 0.043). Again, no significant effect of demonstrator condition was identified. We thus did not detect a difference in ontogenetic onset of con- and allospecific gaze following.

**Fig. 3:**
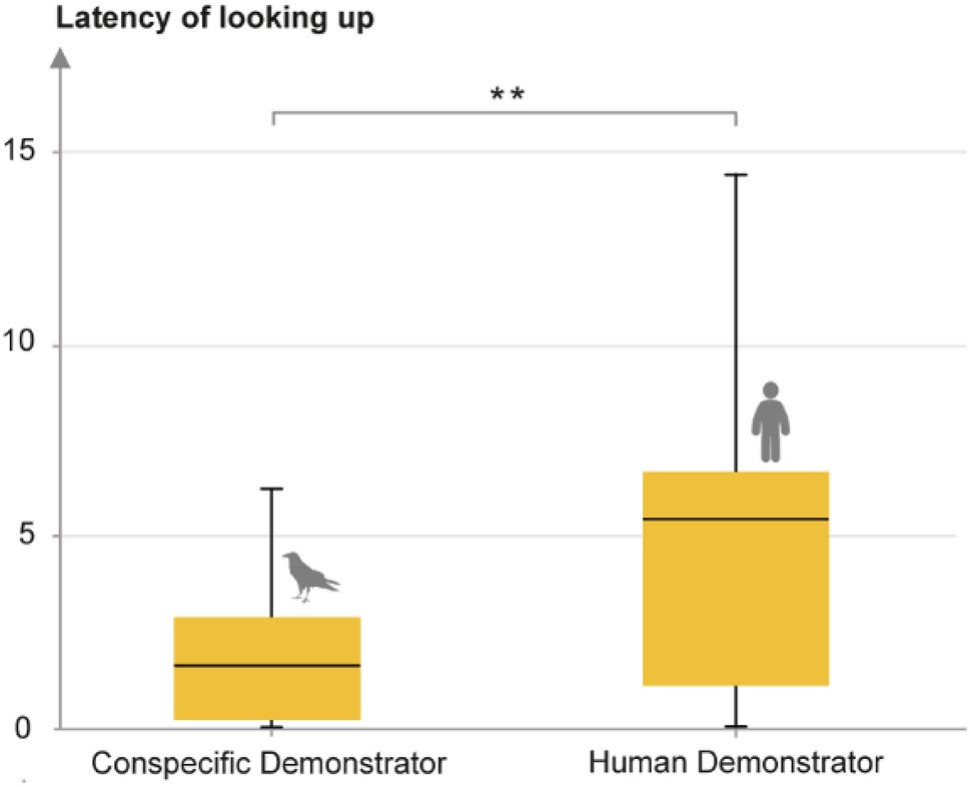
Difference in latency of looking up in test trials with conspecific and human demonstrators

**Fig. 4:**
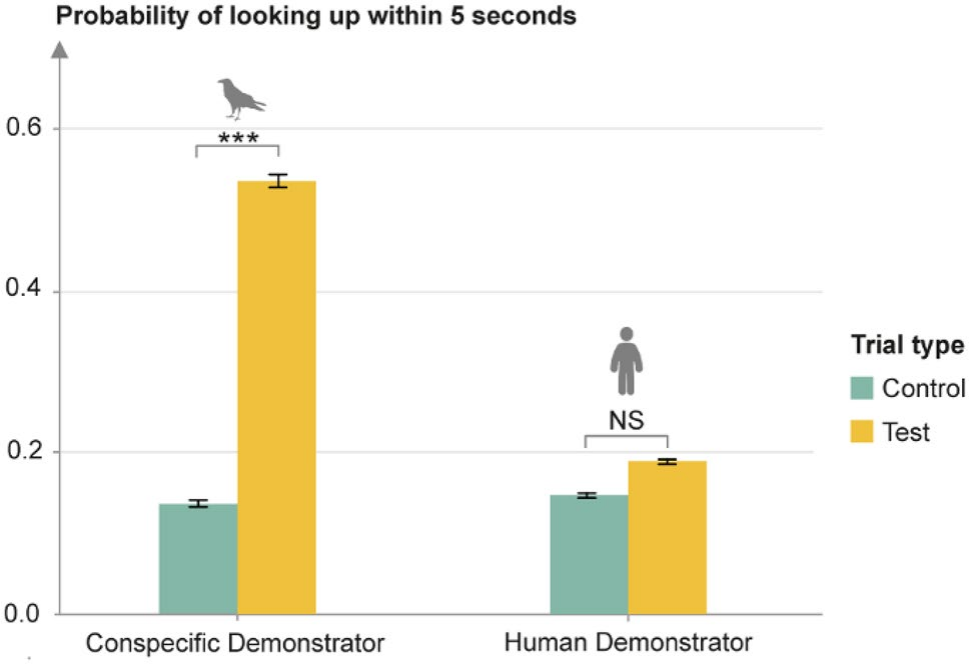
Probability of looking up in control compared to test trials with conspecific and human demonstrators after introducing a 5-s criterion for upward looks to be considered gaze follows

When comparing latencies of co-orientations in test trials, ravens looked up significantly quicker with conspecific demonstrators compared to human demonstrators (GLMM; *χ*^2^ = 8.85, *df* = 1, *p* = 0.0029, see Fig. 3). Based on the difference in mean latencies (1.96 s after the onset of gaze demonstration, i.e., the demonstrator looking up, with conspecifics compared to 4.76 s with humans), we introduced a 5-s cut-off for upward looks to be scored as gaze follows. This revealed a significant effect of the demonstrator condition (GLMM; *χ*^2^ = 5.20, *df* = 1, *p* = 0.023). In fact, when analyzing the demonstrator conditions separately with this new criterion, no significant difference between test and control trials could be identified anymore in the human condition (see Fig. 4). In the conspecific condition, the effect of trial type became even stronger, as the new criterion removed some upward looks from control trials, but none from test trials (GLMM; *χ*^2^ = 13.46, *df* = 1, *p* = 0.00024, see Fig. 4).

All four juvenile ravens “checked back” to the demonstrator after following their gaze and did so already from the onset of the study, i.e., as early as 30 days old. Again, the final model excluded age and explained more variance than when including this factor (Δ AIC > 10). Thus, no developmental effect over the experimental period could be identified. However, a significant effect of demonstrator condition on “checking back” was found (GLMM; *χ*^2^ = 9.28, *df* = 1, *p* = 0.0023). Juvenile ravens checked back significantly more often with conspecific compared to human demonstrators.

## Discussion

We investigated the development of gaze following in juvenile common ravens and the effect of con- and allospecific demonstrators. The ravens in this study already occasionally co-oriented with conspecifics at study onset, but only started to significantly follow the gazes of both human and conspecific demonstrators between 51 and 60 days (7.5–8.5 weeks). We did not detect a difference in the ontogenetic onset of gaze following between the two demonstrator conditions. This could, however, be a methodological artefact due to low sample sizes. Our findings are in line with the results of Bugnyar and colleagues (2004), reporting that ravens first started to follow the gaze of a human demonstrator at 8 weeks.

Only one other study (Schloegl et al. 2007) described the development of conspecific gaze following in ravens and observed first spontaneous visual co-orientations with siblings “a few days after fledging” (Schloegl et al. 2007, p.772). In the present study, we first recorded visual co-orientations with siblings at 30 days old, and thus even before fledging. These co-orientations were, however, not yet occurring on a statistically significant level. True gaze following skills only developed after fledging.

When analyzing upward looks in the full 10 s of the trials, no significant effect of demonstrator condition on overall looking-up rate was identified, indicating co-orientations with both humans and conspecifics. Many species have been found capable of following human gaze, especially primates (for a review see Rosati and Hare 2009). However, even within primates, cotton-top tamarins (*Saguinus oedipus*) only follow the gazes of conspecifics (Neiworth et al. 2002). Chimpanzees follow human gazes, but at significantly lower rates compared to conspecifics (Hattori et al. 2010). And even domesticated ungulates prefer to follow the gaze of a conspecific over that of a human experimenter (Schaffer et al. 2020).

Similarly, after introducing a more conservative criterion for gaze follows in our study, i.e., a 5-s cut-off after the onset of the gaze cue for upward looks to be scored as gaze follows, no significant effect of trial type could be identified any longer with a human demonstrator. Juvenile ravens might hence perhaps not follow human gaze at all in this age range.

Nevertheless, two lines of evidence speak against this. First, without the time cut-off, ravens looked up significantly more often in test compared to control trials with human demonstrators. The only difference between the two trial types was the human gaze cue, suggesting that the gaze caused the difference in upward looks. Second, we found “checking back” behaviour in human test trials, implying that co-orientations with humans were indeed incidences of gaze following.

The prolonged time to react to human gaze could be the result of longer processing times to interpret allospecific gaze. To our knowledge, no study has compared latencies of co-orientation with con- and allospecific demonstrators. One should note that in the human demonstrator condition, the human was gazing continuously for 5 s, and the subject reacted, on average, after 4.76 s. The gaze of a conspecific, though, was a quick spontaneous gaze towards the laser pointer, lasting on average 3.6 s, but with several instances only lasting for 1 s. In other words, such quick gazes by a human would probably not have elicited a response in the young ravens.

Finally, we observed “checking back” in juvenile ravens as young as 30 days old and thus even before fledging. That is very early compared to human infants and great apes (Bräuer et al. 2005; Scaife and Bruner 1975). This finding does not only support the hypothesis that “checking back” is a shared skill among birds, but also implies that birds form social predictions about others exceptionally early in their ontogeny. Studies with higher sample sizes and even earlier onset will be needed to pinpoint the ontogenetic onset of this behaviour.

We, moreover, found a difference in “checking back” rates between demonstrator conditions. Juvenile ravens checked back significantly less with humans compared to conspecifics. This discrepancy might be the result of differences in the formation of social predictions about con- and allospecific demonstrators. The quicker responses indicate that ravens are more attuned to conspecific gaze. They might thus have a stronger expectation to find a target in their line of gaze compared to the gaze of a human. The more robust social prediction might cause more surprise when not finding a gaze target, leading to more “checking back” with conspecifics compared to humans.

There are two alternative explanations for this phenomenon. First, the difference in “checking back” with humans and conspecifics might not be caused by more robust social predictions, but by different predictions for humans and conspecifics. Ravens are food cachers. Consequently, when not finding an object in the line of sight of a conspecific, it could be beneficial to continue the search, while they might not have such predictions for human behaviour. However, it should be noted that ravens only start caching approximately 2 months after fledging (though premature forms of this behaviour can already occur shortly after fledging; Bugnyar et al. 2007)—considerably later than the onset of “checking back” in our study. They, moreover, had never observed adult ravens nor humans cache food. Second, an inherent anatomical difference between humans and ravens, such as the pointy beak, might allow for more accurate tracking of gaze directions and thus more robust predictions about the location of gaze targets. More nuanced studies investigating “checking back” in ravens based on human and conspecific gaze cues will be needed to understand the differences in this behaviour.

Taken together, even though we did not find a difference in the ontogenetic onset of gaze following with con- and allospecific demonstrators, our findings suggest differences in processing speed and “checking back” diagnostic of social predictions between the two. This indicates different ecological, anatomical, or other valences of con- and allospecific gaze—at least for very young individuals.

It should be noted that the subjects of this study were hand-raised by the human experimenters in this study, and thus had ample exposure and positive experiences with humans. One could even argue that the human demonstrators in this study were more important to the survival of the subjects than the conspecifics, as they were food providers. Despite this, we found the above-described differences in the gaze following responses between humans and conspecifics.

This indicates that the neurocognitive mechanisms involved in gaze following are intrinsically attuned to conspecifics, likely because they have evolved to optimize social information gathering within a social group of conspecifics. Gaze following studies using human demonstrators might thus not have discovered species’ full gaze following potentials in terms of speed and rate of co-orientations. Follow-up studies should investigate whether ravens overcome this discrepancy and eventually develop the same gaze following responses towards humans. Indeed, the opposite would be of interest too: do humans note and follow the gazes of ravens to the same extent as ravens do.

Nevertheless, there are disadvantages when using conspecific demonstrators. Length and exact location of gaze cues are less controlled when luring an animal to gaze towards a stimulus. This might, however, make these gazes more realistic and consequently encourage gaze following responses. Due to the above-mentioned advantages and differences in outcomes when using con- and allospecific demonstrators, we propose more studies using conspecific demonstrators to reveal animals’ true gaze following potentials.

## Supplementary Information

Below is the link to the electronic supplementary material.

Supplementary file1: Example video of a human control trial (TS 48574 KB)

Supplementary file2: Example video of a human test trial (TS 61497 KB)

Supplementary file3: Example video of a conspecific control trial (TS 36790 KB)

Supplementary file4: Example video of a conspecific test trial (TS 15939 KB)

Supplementary file5: Example video of a stimulus control trial (TS 45212 KB)

Supplementary file6 (DOCX 85 KB)

## Data Availability

The data for the reported study can be found in the Supplementary Materials (see Supplementary Table 3).
