# Supplementary material for: Differential responses to con- and allospecific visual cues in juvenile ravens (Corvus corax): the ontogeny of gaze following and social predictions
Source: Anim Cogn. 2023 Apr 7;26(4):1251–8. doi: 10.1007/s10071-023-01772-3 (PMC10345056; doi:10.1007/s10071-023-01772-3)
Supplement: Supplementary file 6 — Supplementary file6 (DOCX 85 KB) [file 10071_2023_1772_MOESM6_ESM.docx]

**Supplementary Materials for**

**Differential responses to con- and allospecific visual cues in juvenile ravens (*Corvus corax*): The ontogeny of gaze following and social predictions**

**Animal Cognition**

Claudia Zeiträg* & Mathias Osvath

*Corresponding author. Email: [claudia.zeitrag@lucs.lu.se](mailto:claudia.zeitrag@lucs.lu.se); Department of Philosophy and Cognitive Science, Lund University

**1. Subjects**

Table 1: Test subjects

| **Subject** | **Age at study onset [days]** | **Date of collection** | |  |
| --- | --- | --- | --- | --- |
| Red | 30 | | 210425 | |
| Blue | 37 | | 210426 | |
| Yellow | 44 | | 210425 | |
| White | 44 | | 210425 | |

**2. Trials**

Table 2: Numbers of trials for each subject and condition per experimental session

| Date | Subject | Age [days] | Demonstrator Condition | Test Condition | #Trials |
| --- | --- | --- | --- | --- | --- |
| 210512 | Red | 30 | Conspecific | Test | 2 |
|  | Red | 30 | Conspecific | Control | 2 |
|  | Blue | 37 | Conspecific | Test | 2 |
|  | Blue | 37 | Conspecific | Control | 2 |
|  | Yellow | 44 | Conspecific | Test | 2 |
|  | Yellow | 44 | Conspecific | Control | 2 |
|  | White | 44 | Conspecific | Test | 2 |
|  | White | 44 | Conspecific | Control | 2 |
| 210517 | Red | 35 | Human | Test | 2 |
|  | Red | 35 | Human | Control | 2 |
|  | Red | 35 | Conspecific | Test | 2 |
|  | Red | 35 | Conspecific | Control | 1 |
|  | Blue | 42 | Human | Test | 2 |
|  | Blue | 42 | Human | Control | 2 |
|  | Blue | 42 | Conspecific | Test | 2 |
|  | Blue | 42 | Conspecific | Control | 2 |
|  | Yellow | 49 | Human | Test | 1 |
|  | Yellow | 49 | Human | Control | 1 |
|  | Yellow | 49 | Conspecific | Test | 1 |
|  | Yellow | 49 | Conspecific | Control | 2 |
|  | White | 49 | Human | Test | 2 |
|  | White | 49 | Human | Control | 2 |
|  | White | 49 | Conspecific | Test | 1 |
|  | White | 49 | Conspecific | Control | 1 |
| 210521 | Red | 39 | Human | Test | 2 |
|  | Red | 39 | Human | Control | 2 |
|  | Red | 39 | Conspecific | Test | 2 |
|  | Red | 39 | Conspecific | Control | 2 |
|  | Blue | 46 | Human | Test | 2 |
|  | Blue | 46 | Human | Control | 2 |
|  | Blue | 46 | Conspecific | Test | 2 |
|  | Blue | 46 | Conspecific | Control | 2 |
|  | Yellow | 53 | Human | Test | 2 |
|  | Yellow | 53 | Human | Control | 2 |
|  | Yellow | 53 | Conspecific | Test | 2 |
|  | Yellow | 53 | Conspecific | Control | 2 |
|  | White | 53 | Human | Test | 2 |
|  | White | 53 | Human | Control | 2 |
|  | White | 53 | Conspecific | Test | 2 |
|  | White | 53 | Conspecific | Control | 2 |
| 210524 | Red | 42 | Human | Test | 2 |
|  | Red | 42 | Human | Control | 2 |
|  | Red | 42 | Conspecific | Test | 2 |
|  | Red | 42 | Conspecific | Control | 2 |
|  | Red | 42 | Conspecific | Stimulus Control | 2 |
|  | Blue | 49 | Human | Test | 2 |
|  | Blue | 49 | Human | Control | 2 |
|  | Blue | 49 | Conspecific | Test | 2 |
|  | Blue | 49 | Conspecific | Control | 2 |
|  | Blue | 49 | Conspecific | Stimulus Control | 1 |
|  | Yellow | 56 | Human | Test | 2 |
|  | Yellow | 56 | Human | Control | 2 |
|  | Yellow | 56 | Conspecific | Test | 2 |
|  | Yellow | 56 | Conspecific | Control | 2 |
|  | Yellow | 56 | Conspecific | Stimulus Control | 2 |
|  | White | 56 | Human | Test | 2 |
|  | White | 56 | Human | Control | 2 |
|  | White | 56 | Conspecific | Test | 2 |
|  | White | 56 | Conspecific | Control | 2 |
|  | White | 56 | Conspecific | Stimulus Control | 2 |
| 210528 | Red | 46 | Human | Test | 2 |
|  | Red | 46 | Human | Control | 2 |
|  | Red | 46 | Conspecific | Test | 2 |
|  | Red | 46 | Conspecific | Control | 2 |
|  | Red | 46 | Conspecific | Stimulus Control | 2 |
|  | Blue | 53 | Human | Test | 2 |
|  | Blue | 53 | Human | Control | 2 |
|  | Blue | 53 | Conspecific | Test | 2 |
|  | Blue | 53 | Conspecific | Control | 2 |
|  | Blue | 53 | Conspecific | Stimulus Control | 2 |
|  | Yellow | 60 | Human | Test | 2 |
|  | Yellow | 60 | Human | Control | 2 |
|  | Yellow | 60 | Conspecific | Test | 2 |
|  | Yellow | 60 | Conspecific | Control | 2 |
|  | Yellow | 60 | Conspecific | Stimulus Control | 2 |
|  | White | 60 | Human | Test | 2 |
|  | White | 60 | Human | Control | 2 |
|  | White | 60 | Conspecific | Test | 2 |
|  | White | 60 | Conspecific | Control | 2 |
|  | White | 60 | Conspecific | Stimulus Control | 2 |
| 210531 | Red | 49 | Human | Test | 2 |
|  | Red | 49 | Human | Control | 2 |
|  | Red | 49 | Conspecific | Control | 1 |
|  | Red | 49 | Conspecific | Stimulus Control | 2 |
|  | Blue | 56 | Human | Test | 1 |
|  | Blue | 56 | Human | Control | 2 |
|  | Blue | 56 | Conspecific | Test | 1 |
|  | Blue | 56 | Conspecific | Control | 1 |
|  | Blue | 56 | Conspecific | Stimulus Control | 2 |
|  | Yellow | 63 | Human | Test | 2 |
|  | Yellow | 63 | Human | Control | 2 |
|  | Yellow | 63 | Conspecific | Test | 1 |
|  | Yellow | 63 | Conspecific | Stimulus Control | 2 |
|  | White | 63 | Human | Test | 1 |
|  | White | 63 | Conspecific | Test | 1 |
|  | White | 63 | Conspecific | Control | 2 |
|  | White | 63 | Conspecific | Stimulus Control | 1 |
| 210607 | Red | 56 | Human | Test | 2 |
|  | Red | 56 | Human | Control | 2 |
|  | Red | 56 | Conspecific | Stimulus Control | 2 |
|  | Blue | 63 | Human | Test | 2 |
|  | Blue | 63 | Human | Control | 2 |
|  | Blue | 63 | Conspecific | Stimulus Control | 2 |
|  | Yellow | 70 | Human | Test | 2 |
|  | Yellow | 70 | Human | Control | 2 |
|  | Yellow | 70 | Conspecific | Stimulus Control | 2 |
|  | White | 70 | Human | Test | 2 |
|  | White | 70 | Human | Control | 2 |
|  | White | 70 | Conspecific | Stimulus Control | 2 |
| 210614 | Red | 56 | Human | Test | 2 |
|  | Red | 56 | Human | Control | 2 |
|  | Blue | 70 | Human | Test | 2 |
|  | Blue | 70 | Human | Control | 2 |
|  | Yellow | 77 | Human | Test | 2 |
|  | Yellow | 77 | Human | Control | 2 |
|  | White | 77 | Human | Test | 2 |
|  | White | 77 | Human | Control | 2 |
| 210621 | Red | 70 | Human | Test | 2 |
|  | Red | 70 | Human | Control | 2 |
|  | Blue | 77 | Human | Test | 2 |
|  | Blue | 77 | Human | Control | 2 |
| 210628 | Red | 77 | Human | Test | 1 |
|  | Blue | 84 | Human | Test | 2 |
|  | Blue | 84 | Human | Control | 2 |
|  | White | 91 | Human | Test | 2 |
|  | White | 91 | Human | Control | 2 |
| 210702 | Red | 77 | Human | Test | 1 |
|  | Yellow | 95 | Human | Test | 1 |
|  | Yellow | 95 | Human | Control | 1 |

**3. Coding definitions**

When coding trials, we first specified subject, demonstrator condition (human or conspecific), and trial type (control, test, stimulus control). In control trials and stimulus controls, the subject’s behaviour was coded for 15 seconds, and in test trials for 10 seconds after the demonstration. We coded all upward looks, inferred from beak orientation (lifting beak up) and head orientation (tilting the head to orient one eye to the sky), including latency from the onset of demonstration and duration of visual orientation. We moreover coded “checking back”, defined as a bird co-orienting with the observed gaze direction and looking back to the demonstrator. Again, we coded latency and duration of this behaviour.

**4. Example videos**

Video 1: Example video of trial type Human Control

Video 2: Example video of trial type Human Test

Video 3: Example video of trial type Conspecific Control

Video 4: Example video of trial type Conspecific Test

Video 5: Example video of trial type Stimulus Control

**5. Data**

Table 3: Data of the present study. VCO = visual co-orientation, CB = checking back

| **Date** | **Subject** | **Age [days]** | **Age range** | **Demonstrator Condition** | **Trial Type** | **VCO** | **CB** | **latency VCO** | **latency CB** |
| --- | --- | --- | --- | --- | --- | --- | --- | --- | --- |
| 210512 | White | 44 | 2 | Conspecific | Test | 1 | 0 | 2.6 | NA |
| 210512 | Blue | 37 | 1 | Conspecific | Control | 1 | NA | 7.4 | NA |
| 210512 | Blue | 37 | 1 | Conspecific | Test | 1 | 1 | 3.8 | 7.4 |
| 210512 | White | 44 | 2 | Conspecific | Control | 0 | NA | NA | NA |
| 210512 | Red | 30 | 1 | Conspecific | Control | 0 | NA | NA | NA |
| 210512 | Red | 30 | 1 | Conspecific | Test | 1 | 1 | 2.4 | 4 |
| 210512 | Blue | 37 | 1 | Conspecific | Test | 1 | 1 | 0 | 3.6 |
| 210512 | Blue | 37 | 1 | Conspecific | Control | 1 | NA | 2 | NA |
| 210512 | Red | 30 | 1 | Conspecific | Test | 1 | 1 | 0.8 | 4.8 |
| 210512 | Red | 30 | 1 | Conspecific | Control | 0 | NA | NA | NA |
| 210512 | Yellow | 44 | 2 | Conspecific | Test | 0 | 0 | NA | NA |
| 210512 | Yellow | 44 | 2 | Conspecific | Control | 0 | NA | NA | NA |
| 210512 | Yellow | 44 | 2 | Conspecific | Control | 0 | NA | NA | NA |
| 210512 | Yellow | 44 | 2 | Conspecific | Test | 1 | 1 | 0.8 | 2 |
| 210512 | White | 44 | 2 | Conspecific | Control | 1 | NA | 1.6 | NA |
| 210512 | White | 44 | 2 | Conspecific | Test | 0 | 0 | NA | NA |
| 210517 | Yellow | 49 | 2 | Conspecific | Control | 0 | NA | NA | NA |
| 210517 | Red | 35 | 1 | Human | Test | 0 | 0 | NA | NA |
| 210517 | Red | 35 | 1 | Human | Control | 0 | NA | NA | NA |
| 210517 | Red | 35 | 1 | Human | Test | 1 | 1 | 0.4 | 4.4 |
| 210517 | Red | 35 | 1 | Human | Control | 0 | NA | NA | NA |
| 210517 | Blue | 42 | 2 | Human | Test | 0 | 0 | NA | NA |
| 210517 | Blue | 42 | 2 | Human | Control | 0 | NA | NA | NA |
| 210517 | Blue | 42 | 2 | Conspecific | Test | 1 | 1 | 1.4 | 12.6 |
| 210517 | Blue | 42 | 2 | Conspecific | Control | 1 | NA | 9 | NA |
| 210517 | Yellow | 49 | 2 | Conspecific | Control | 1 | NA | 2.4 | NA |
| 210517 | Yellow | 49 | 2 | Conspecific | Test | 0 | 0 | NA | NA |
| 210517 | White | 49 | 2 | Conspecific | Control | 0 | NA | NA | NA |
| 210517 | Yellow | 49 | 2 | Human | Control | 0 | NA | NA | NA |
| 210517 | White | 49 | 2 | Conspecific | Test | 1 | 1 | 1.6 | 3 |
| 210517 | Blue | 42 | 2 | Conspecific | Test | 1 | 1 | 0.2 | 4 |
| 210517 | Blue | 42 | 2 | Conspecific | Control | 0 | NA | NA | NA |
| 210517 | Red | 35 | 2 | Conspecific | Test | 0 | 0 | NA | NA |
| 210517 | Red | 35 | 2 | Conspecific | Control | 0 | NA | NA | NA |
| 210517 | Blue | 42 | 2 | Human | Test | 0 | 0 | NA | NA |
| 210517 | Blue | 42 | 2 | Human | Control | 0 | NA | NA | NA |
| 210517 | Red | 35 | 2 | Conspecific | Test | 0 | 0 | NA | NA |
| 210517 | White | 49 | 2 | Human | Test | 1 | 0 | 14.4 | NA |
| 210517 | White | 49 | 2 | Human | Test | 0 | 0 | NA | NA |
| 210517 | White | 49 | 2 | Human | Control | 0 | NA | NA | NA |
| 210517 | Yellow | 49 | 2 | Human | Test | 1 | 0 | 6.6 | NA |
| 210517 | White | 49 | 2 | Human | Control | 0 | NA | NA | NA |
| 210521 | Red | 39 | 2 | Human | Control | 0 | NA | NA | NA |
| 210521 | Red | 39 | 2 | Human | Test | 0 | 0 | NA | NA |
| 210521 | Red | 39 | 2 | Human | Test | 0 | 0 | NA | NA |
| 210521 | Blue | 46 | 2 | Conspecific | Control | 0 | NA | NA | NA |
| 210521 | Yellow | 53 | 3 | Human | Control | 0 | NA | NA | NA |
| 210521 | Yellow | 53 | 3 | Human | Test | 1 | 0 | 6 | NA |
| 210521 | White | 53 | 3 | Human | Test | 1 | 0 | 5.4 | NA |
| 210521 | White | 53 | 3 | Human | Test | 0 | 0 | NA | NA |
| 210521 | Red | 39 | 2 | Conspecific | Control | 0 | NA | NA | NA |
| 210521 | Red | 39 | 2 | Conspecific | Control | 0 | NA | NA | NA |
| 210521 | Red | 39 | 2 | Conspecific | Test | 0 | 0 | NA | NA |
| 210521 | Yellow | 53 | 3 | Human | Control | 1 | NA | 0.2 | NA |
| 210521 | Yellow | 53 | 3 | Human | Test | 0 | 0 | NA | NA |
| 210521 | Red | 39 | 2 | Conspecific | Test | 1 | 1 | 2 | 3.2 |
| 210521 | Red | 39 | 2 | Human | Control | 0 | NA | NA | NA |
| 210521 | White | 53 | 3 | Conspecific | Test | 1 | 1 | 0.2 | 1.4 |
| 210521 | White | 53 | 3 | Conspecific | Test | 0 | 0 | NA | NA |
| 210521 | White | 53 | 3 | Conspecific | Control | 1 | NA | 0.8 | NA |
| 210521 | White | 53 | 3 | Conspecific | Control | 0 | NA | NA | NA |
| 210521 | Yellow | 53 | 3 | Conspecific | Test | 1 | 1 | 3.8 | 4.4 |
| 210521 | Yellow | 53 | 3 | Conspecific | Test | 0 | 0 | NA | NA |
| 210521 | Yellow | 53 | 3 | Conspecific | Control | 0 | NA | NA | NA |
| 210521 | Yellow | 53 | 3 | Conspecific | Control | 0 | NA | NA | NA |
| 210521 | Blue | 46 | 2 | Human | Control | 1 | NA | 3 | NA |
| 210521 | Blue | 46 | 2 | Human | Test | 0 | 0 | NA | NA |
| 210521 | Blue | 46 | 2 | Human | Test | 1 | 1 | 5.6 | 6.4 |
| 210521 | Blue | 46 | 2 | Human | Control | 0 | NA | NA | NA |
| 210521 | White | 53 | 3 | Human | Control | 1 | NA | 1.4 | NA |
| 210521 | White | 53 | 3 | Human | Control | 1 | NA | 4 | NA |
| 210521 | Blue | 46 | 2 | Conspecific | Test | 0 | 0 | NA | NA |
| 210521 | Blue | 46 | 2 | Conspecific | Test | 0 | 0 | NA | NA |
| 210521 | Blue | 46 | 2 | Conspecific | Control | 0 | NA | NA | NA |
| 210524 | Blue | 49 | 2 | Conspecific | Test | 0 | 0 | NA | NA |
| 210524 | Blue | 49 | 2 | Conspecific | Control | 0 | NA | NA | NA |
| 210524 | Blue | 49 | 2 | Conspecific | Test | 1 | 1 | 0 | 3.4 |
| 210524 | Blue | 49 | 2 | Conspecific | Control | 0 | NA | NA | NA |
| 210524 | Yellow | 56 | 3 | Human | Test | 1 | 1 | 0.2 | 0.6 |
| 210524 | White | 56 | 3 | Conspecific | Test | 1 | 1 | 2.4 | 3.4 |
| 210524 | White | 56 | 3 | Conspecific | Control | 0 | NA | NA | NA |
| 210524 | White | 56 | 3 | Conspecific | Control | 0 | NA | NA | NA |
| 210524 | White | 56 | 3 | Conspecific | Test | 1 | 1 | 2.4 | 8.6 |
| 210524 | White | 56 | 3 | Human | Test | 0 | 0 | NA | NA |
| 210524 | White | 56 | 3 | Human | Control | 1 | NA | 3.2 | NA |
| 210524 | White | 56 | 3 | Human | Control | 0 | NA | NA | NA |
| 210524 | White | 56 | 3 | Human | Test | 0 | 0 | NA | NA |
| 210524 | Yellow | 56 | 3 | Conspecific | Test | 0 | 0 | NA | NA |
| 210524 | Yellow | 56 | 3 | Conspecific | Test | 1 | 1 | 5 | 8.6 |
| 210524 | Yellow | 56 | 3 | Conspecific | Control | 0 | NA | NA | NA |
| 210524 | White | 56 | 3 | Conspecific | Stimulus Control | 1 | NA | 0.8 | NA |
| 210524 | Yellow | 56 | 3 | Conspecific | Stimulus Control | 0 | NA | NA | NA |
| 210524 | Yellow | 56 | 3 | Human | Test | 1 | 0 | 0.6 | NA |
| 210524 | Yellow | 56 | 3 | Conspecific | Stimulus Control | 1 | NA | 2.4 | NA |
| 210524 | Yellow | 56 | 3 | Human | Control | 0 | NA | NA | NA |
| 210524 | Yellow | 56 | 3 | Human | Control | 0 | NA | NA | NA |
| 210524 | White | 56 | 3 | Conspecific | Stimulus Control | 0 | NA | NA | NA |
| 210524 | Yellow | 56 | 3 | Conspecific | Control | 1 | NA | 2.4 | NA |
| 210524 | Red | 42 | 2 | Human | Test | 0 | 0 | NA | NA |
| 210524 | Red | 42 | 2 | Conspecific | Stimulus Control | 0 | NA | NA | NA |
| 210524 | Red | 42 | 2 | Conspecific | Stimulus Control | 0 | NA | NA | NA |
| 210524 | Red | 42 | 2 | Human | Test | 0 | 0 | NA | NA |
| 210524 | Red | 42 | 2 | Human | Control | 0 | NA | NA | NA |
| 210524 | Red | 42 | 2 | Human | Control | 0 | NA | NA | NA |
| 210524 | Red | 42 | 2 | Conspecific | Test | 0 | 0 | NA | NA |
| 210524 | Red | 42 | 2 | Conspecific | Test | 0 | 0 | NA | NA |
| 210524 | Red | 42 | 2 | Conspecific | Control | 0 | NA | NA | NA |
| 210524 | Red | 42 | 2 | Conspecific | Control | 0 | NA | NA | NA |
| 210524 | Blue | 49 | 2 | Human | Test | 0 | 0 | NA | NA |
| 210524 | Blue | 49 | 2 | Conspecific | Stimulus Control | 0 | NA | NA | NA |
| 210524 | Blue | 49 | 2 | Human | Control | 0 | NA | NA | NA |
| 210524 | Blue | 49 | 2 | Human | Test | 0 | 0 | NA |  |
| 210524 | Blue | 49 | 2 | Human | Control | 0 | NA | NA | NA |
| 210524 | Blue | 49 | 2 | Human | Control | 0 | NA | NA | NA |
| 210528 | Blue | 53 | 3 | Conspecific | Control | 0 | NA | NA | NA |
| 210528 | Blue | 53 | 3 | Conspecific | Test | 1 | 1 | 3.6 | 7.2 |
| 210528 | Blue | 53 | 3 | Conspecific | Test | 1 | 1 | 1.6 | 13.6 |
| 210528 | Blue | 53 | 3 | Conspecific | Control | 0 | NA | NA | NA |
| 210528 | Red | 46 | 2 | Conspecific | Test | 1 | 1 | 0 | 0.2 |
| 210528 | Red | 46 | 2 | Conspecific | Control | 0 | NA | NA | NA |
| 210528 | Blue | 53 | 3 | Human | Test | 0 | 0 | NA | NA |
| 210528 | Blue | 53 | 3 | Human | Test | 0 | 0 | NA | NA |
| 210528 | Blue | 53 | 3 | Conspecific | Stimulus Control | 0 | NA | NA | NA |
| 210528 | Red | 46 | 2 | Human | Test | 0 | 0 | NA | NA |
| 210528 | Red | 46 | 2 | Conspecific | Stimulus Control | 0 | NA | NA | NA |
| 210528 | Red | 46 | 2 | Human | Control | 0 | NA | NA | NA |
| 210528 | Red | 46 | 2 | Human | Test | 0 | 0 | NA | NA |
| 210528 | Red | 46 | 2 | Conspecific | Stimulus Control | 0 | NA | NA | NA |
| 210528 | Red | 46 | 2 | Human | Control | 0 | NA | NA | NA |
| 210528 | White | 60 | 3 | Conspecific | Control | 0 | NA | NA | NA |
| 210528 | White | 60 | 3 | Conspecific | Test | 1 | 1 | 0.2 | 2 |
| 210528 | White | 60 | 3 | Conspecific | Control | 0 | NA | NA | NA |
| 210528 | White | 60 | 3 | Conspecific | Test | 1 | 0 | 0 | NA |
| 210528 | Blue | 53 | 3 | Human | Control | 1 | NA | 3.4 | NA |
| 210528 | Blue | 53 | 3 | Human | Control | 1 | NA | 1.6 | NA |
| 210528 | White | 60 | 3 | Conspecific | Stimulus Control | 1 | NA | 14.4 | NA |
| 210528 | White | 60 | 3 | Human | Control | 0 | NA | NA | NA |
| 210528 | White | 60 | 3 | Human | Test | 1 | 0 | 6.6 | NA |
| 210528 | White | 60 | 3 | Conspecific | Stimulus Control | 0 | NA | NA | NA |
| 210528 | Blue | 53 | 3 | Conspecific | Stimulus Control | 1 | NA | 0.4 | NA |
| 210528 | White | 60 | 3 | Human | Control | 1 | NA | 2.4 | NA |
| 210528 | White | 60 | 3 | Human | Test | 1 | 1 | 5.6 | 11.4 |
| 210528 | Yellow | 60 | 3 | Human | Test | 1 | 0 | 4.6 | NA |
| 210528 | Yellow | 60 | 3 | Conspecific | Stimulus Control | 1 | NA | 2.4 | NA |
| 210528 | Yellow | 60 | 3 | Human | Test | 0 | 0 | NA | NA |
| 210528 | Yellow | 60 | 3 | Human | Control | 0 | NA | NA | NA |
| 210528 | Yellow | 60 | 3 | Human | Control | 1 | NA | 5.8 | NA |
| 210528 | Yellow | 60 | 3 | Conspecific | Stimulus Control | 0 | NA | NA | NA |
| 210528 | Yellow | 60 | 3 | Conspecific | Test | 1 | 1 | 1.2 | 2 |
| 210528 | Yellow | 60 | 3 | Conspecific | Test | 0 | 0 | NA | NA |
| 210528 | Yellow | 60 | 3 | Conspecific | Control | 0 | NA | NA | NA |
| 210528 | Yellow | 60 | 3 | Conspecific | Control | 0 | NA | NA | NA |
| 210528 | Red | 46 | 2 | Conspecific | Test | 0 | 0 | NA | NA |
| 210528 | Red | 46 | 2 | Conspecific | Control | 0 | NA | NA | NA |
| 210531 | White | 63 | 4 | Conspecific | Test | 0 | 0 | NA | NA |
| 210531 | White | 63 | 4 | Conspecific | Control | 1 | NA | 1.8 | NA |
| 210531 | Blue | 56 | 3 | Human | Test | 1 | 0 | 0 | NA |
| 210531 | Blue | 56 | 3 | Conspecific | Stimulus Control | 0 | NA | NA | NA |
| 210531 | Blue | 56 | 3 | Conspecific | Stimulus Control | 1 | NA | 0.2 | NA |
| 210531 | Blue | 56 | 3 | Human | Control | 0 | NA | NA | NA |
| 210531 | Yellow | 63 | 4 | Conspecific | Stimulus Control | 0 | NA | NA | NA |
| 210531 | Yellow | 63 | 4 | Human | Test | 1 | 1 | 1.6 | 4.2 |
| 210531 | Yellow | 63 | 4 | Human | Test | 1 | 0 | 0.6 | NA |
| 210531 | Yellow | 63 | 4 | Human | Control | 0 | NA | NA | NA |
| 210531 | Blue | 56 | 3 | Conspecific | Control | 0 | NA | NA | NA |
| 210531 | Blue | 56 | 3 | Conspecific | Test | 0 | 0 | NA | NA |
| 210531 | Red | 49 | 2 | Conspecific | Control | 0 | NA | NA | NA |
| 210531 | Yellow | 63 | 4 | Conspecific | Test | 1 | 0 | 6.2 | NA |
| 210531 | Yellow | 63 | 4 | Conspecific | Stimulus Control | 0 | NA | NA | NA |
| 210531 | Blue | 56 | 3 | Human | Control | 1 | NA | 5.2 | NA |
| 210531 | Yellow | 63 | 4 | Human | Control | 0 | NA | NA | NA |
| 210531 | Red | 49 | 2 | Human | Control | 0 | NA | NA | NA |
| 210531 | Red | 49 | 2 | Human | Test | 1 | 1 | 0.6 | 3.8 |
| 210531 | Red | 49 | 2 | Conspecific | Stimulus Control | 1 | NA | 9.6 | NA |
| 210531 | Red | 49 | 2 | Human | Control | 0 | NA | NA | NA |
| 210531 | Red | 49 | 2 | Human | Test | 0 | 0 | NA | NA |
| 210531 | Red | 49 | 2 | Conspecific | Stimulus Control | 0 | NA | NA | NA |
| 210531 | White | 63 | 4 | Conspecific | Control | 0 | NA | NA | NA |
| 210531 | White | 63 | 4 | Human | Test | 0 | 0 | NA | NA |
| 210531 | White | 63 | 4 | Conspecific | Stimulus Control | 0 | NA | NA | NA |
| 210604 | Red | 53 | 3 | Conspecific | Stimulus Control | 0 | NA | NA | NA |
| 210604 | Red | 53 | 3 | Human | Control | 0 | NA | NA | NA |
| 210604 | Red | 53 | 3 | Human | Test | 0 | 0 | NA | NA |
| 210604 | Red | 53 | 3 | Human | Control | 0 | NA | NA | NA |
| 210604 | Red | 53 | 3 | Conspecific | Stimulus Control | 0 | NA | NA | NA |
| 210604 | Red | 53 | 3 | Human | Test | 0 | 0 | NA | NA |
| 210604 | White | 67 | 4 | Conspecific | Test | 1 | 1 | 4.8 | 8.2 |
| 210604 | White | 67 | 4 | Conspecific | Test | 0 | 0 | NA | NA |
| 210604 | Yellow | 67 | 4 | Human | Control | 1 | NA | 2.8 | NA |
| 210604 | Yellow | 67 | 4 | Conspecific | Stimulus Control | 0 | NA | NA | NA |
| 210604 | Yellow | 67 | 4 | Human | Test | 0 | 0 | NA | NA |
| 210604 | Blue | 60 | 3 | Human | Test | 1 | 1 | 0 | 1.2 |
| 210604 | White | 67 | 4 | Conspecific | Control | 1 | NA | 5.4 | NA |
| 210604 | White | 67 | 4 | Conspecific | Control | 0 | NA | NA | NA |
| 210604 | Blue | 60 | 3 | Conspecific | Stimulus Control | 1 | NA | 3.8 | NA |
| 210604 | Yellow | 67 | 4 | Human | Control | 1 | NA | 7.8 | NA |
| 210604 | Yellow | 67 | 4 | Human | Test | 1 | 1 | 3.4 | 7.8 |
| 210604 | Yellow | 67 | 4 | Conspecific | Stimulus Control | 0 | NA | NA | NA |
| 210607 | Blue | 63 | 4 | Human | Control | 0 | NA | NA | NA |
| 210607 | Blue | 63 | 4 | Human | Control | 0 | NA | NA | NA |
| 210607 | Blue | 63 | 4 | Conspecific | Stimulus Control | 0 | NA | NA | NA |
| 210607 | Blue | 63 | 4 | Conspecific | Stimulus Control | 1 | NA | 13.8 | NA |
| 210607 | Yellow | 70 | 4 | Human | Test | 0 | 0 | NA | NA |
| 210607 | Yellow | 70 | 4 | Human | Control | 1 | NA | 0.2 | NA |
| 210607 | Yellow | 70 | 4 | Conspecific | Stimulus Control | 0 | NA | NA | NA |
| 210607 | Yellow | 70 | 4 | Conspecific | Stimulus Control | 1 | NA | 6.2 | NA |
| 210607 | Yellow | 70 | 4 | Human | Test | 1 | 1 | 5.8 | 7.4 |
| 210607 | Yellow | 70 | 4 | Human | Control | 0 | NA | NA | NA |
| 210607 | Red | 56 | 3 | Human | Test | 1 | 0 | 2.6 | NA |
| 210607 | Red | 56 | 3 | Human | Test | 0 | 0 | NA | NA |
| 210607 | Red | 56 | 3 | Conspecific | Stimulus Control | 0 | NA | NA | NA |
| 210607 | Red | 56 | 3 | Conspecific | Stimulus Control | 0 | NA | NA | NA |
| 210607 | Red | 56 | 3 | Human | Control | 0 | NA | NA | NA |
| 210607 | White | 70 | 4 | Conspecific | Stimulus Control | 0 | NA | NA | NA |
| 210607 | White | 70 | 4 | Human | Test | 1 | 1 | 6.2 | 11 |
| 210607 | White | 70 | 4 | Human | Control | 0 | NA | NA | NA |
| 210607 | Red | 56 | 3 | Human | Control | 0 | NA | NA | NA |
| 210607 | White | 70 | 4 | Human | Control | 0 | NA | NA | NA |
| 210607 | White | 70 | 4 | Human | Test | 0 | 0 | NA | NA |
| 210607 | White | 70 | 4 | Conspecific | Stimulus Control | 0 | NA | NA | NA |
| 210607 | Blue | 63 | 4 | Human | Test | 0 | 0 | NA | NA |
| 210607 | Blue | 63 | 4 | Human | Test | 0 | 0 | NA | NA |
| 210614 | Blue | 70 | 4 | Human | Control | 0 | NA | NA | NA |
| 210614 | Blue | 70 | 4 | Human | Test | 1 | 0 | 7.4 | NA |
| 210614 | Blue | 70 | 4 | Human | Test | 0 | 0 | NA | NA |
| 210614 | Blue | 70 | 4 | Human | Control | 0 | NA | NA | NA |
| 210614 | Red | 63 | 4 | Human | Test | 0 | 0 | NA | NA |
| 210614 | Red | 63 | 4 | Human | Control | 0 | NA | NA | NA |
| 210614 | Red | 63 | 4 | Human | Test | 1 | 1 | 8.4 | 12.2 |
| 210614 | Red | 63 | 4 | Human | Control | 0 | NA | NA | NA |
| 210614 | White | 77 | 5 | Human | Control | 0 | NA | NA | NA |
| 210614 | White | 77 | 5 | Human | Test | 0 | 0 | NA | NA |
| 210614 | White | 77 | 5 | Human | Test | 1 | 1 | 9.8 | 14 |
| 210614 | Yellow | 77 | 5 | Human | Control | 0 | NA | NA | NA |
| 210614 | Yellow | 77 | 5 | Human | Control | 0 | NA | NA | NA |
| 210614 | Yellow | 77 | 5 | Human | Test | 0 | 0 | NA | NA |
| 210614 | White | 77 | 5 | Human | Control | 0 | NA | NA | NA |
| 210614 | Yellow | 77 | 5 | Human | Test | 0 | 0 | NA | NA |
| 210621 | Blue | 77 | 5 | Human | Control | 1 | NA | 5.2 | NA |
| 210621 | Blue | 77 | 5 | Human | Test | 0 | 0 | NA | NA |
| 210621 | Blue | 77 | 5 | Human | Test | 0 | 0 | NA | NA |
| 210621 | Blue | 77 | 5 | Human | Control | 0 | NA | NA | NA |
| 210621 | Red | 70 | 4 | Human | Test | 0 | 0 | NA | NA |
| 210621 | Red | 70 | 4 | Human | Control | 0 | NA | NA | NA |
| 210621 | Red | 70 | 4 | Human | Control | 0 | NA | NA | NA |
| 210621 | Red | 70 | 4 | Human | Test | 0 | 0 | NA | NA |
| 210624 | White | 87 | 6 | Human | Test | 0 | 0 | NA | NA |
| 210628 | White | 91 | 7 | Human | Control | 0 | NA | NA | NA |
| 210628 | Blue | 84 | 6 | Human | Test | 1 | NA | 3.2 | NA |
| 210628 | Blue | 84 | 6 | Human | Control | 0 | NA | NA | NA |
| 210628 | Blue | 84 | 6 | Human | Test | 0 | 0 | NA | NA |
| 210628 | Blue | 84 | 6 | Human | Control | 0 | NA | NA | NA |
| 210628 | Red | 77 | 5 | Human | Control | 1 | NA | 7.4 | NA |
| 210628 | White | 91 | 7 | Human | Test | 1 | 1 | 11 | 11.6 |
| 210628 | White | 91 | 7 | Human | Test | 1 | 1 | 8.8 | 11.2 |
| 210628 | White | 91 | 7 | Human | Control | 0 | NA | NA | NA |
| 210702 | Red | 77 | 5 | Human | Test | 0 | 0 | NA | NA |
| 210702 | Yellow | 95 | 7 | Human | Test | 1 | 0 | 3.2 | NA |
| 210702 | Yellow | 95 | 7 | Human | Control | 0 | NA | NA | NA |

**6. Results of statistical analysis**

Table 4: Results of likelihood ratio test performed on the final Generalized Linear Mixed Models (lowest AIC). VCO = visual co-orientation; CB = checking back.

| **Model** | **Response variable** | **Distribution** | **Coefficient** | **Chisq** | **df** | **p** |
| --- | --- | --- | --- | --- | --- | --- |
| Gaze following responses | VCO | Binomial | Trial Type | 12.51 | 2 | 0.0019** |
|  |  |  | Demonstrator Condition | 0.10 | 1 | 0.75 |
|  |  |  | Trial Type*Demonstrator Condition | 1.71 | 1 | 0.19 |
| Latency of gaze following | Latency VCO | Gamma | Demonstrator Condition | 8.85 | 1 | 0.0029** |
| Ontogeny of gaze following; age range 3 | VCO | Binomial | Trial Type  Demonstrator Condition | 6.31  0.040 | 2  1 | 0.043*  0.84 |
| 5-second cut-off for gaze following responses | VCO | Binomial | Trial Type  Demonstrator Condition  Trial Type*Demonstrator Condition | 17.79  0.071  5.96 | 2  1  1 | 0.00014***  0.79  0.015 |
| 5-second cut-off; conspecific condition alone | VCO | Binomial | Trial Type | 17.95 | 2 | 0.00013*** |
| Checking back | CB | Binomial | Demonstrator Condition | 9.28 | 1 | 0.0023 ** |
